# Effectiveness of a prevention program in the incidence of cardiovascular outcomes in a low-income population from Colombia: a real-world propensity score-matched cohort study

**DOI:** 10.1186/s12889-020-09516-5

**Published:** 2020-09-17

**Authors:** Pablo Miranda-Machado, Fernando Salcedo-Mejía, Devian Parra-Padilla, Rusvelt Vargas-Moranth, Nelson Rafael Alvis-Zakzuk, Justo Paz-Wilches, Juan Fernández-Mercado, Fernando De la-Hoz Restrepo, Nelson Alvis-Guzmán

**Affiliations:** 1 ALZAK Foundation, Crespo St 67 #5-44 Cartagena, Colombia; 2 Health Economics Research Group, University of Cartagena, Cartagena, Colombia; 3 Health Risk Management Department, Mutual SER E.P.S, Cartagena, Colombia; 4 Epidemiology and Public Health Evaluation Research Group, National University of Colombia, Bogotá, Colombia

**Keywords:** Cardiovascular risk management program, Cardiovascular outcomes, High blood pressure, Diabetes mellitus, Underserved population

## Abstract

**Background:**

Cardiovascular diseases (CVDs) and diabetes mellitus (DM) are among the leading cause of morbidity and mortality in low-and-middle-income countries (LMICs) but evidence in these contexts regarding the effectiveness of primary prevention interventions taking into account patient adherence is scarce. We aimed to evaluate the effectiveness of a cardiovascular risk management program (De Todo Corazón - DTC program) in the incidence of the first cardiovascular outcome (CVO) in a low-income population from the Caribbean region of Colombia using adherence as the main variable of exposure.

**Methods:**

A retrospective propensity score-matched cohort study was conducted. Adult patients with a diagnosis of hypertension (HTA), diabetes mellitus (DM), chronic kidney disease (CKD), or dyslipidemia affiliated to the DTC program between 2013 and 2018 were considered as the study population. Patients with 30 to 76 years, without a history of CVOs, and with more than 6 months of exposure to the program were included. The main outcome of interest was the reduction in the risk of CVOs (stroke, myocardial infarction, or congestive heart failure) based on the adherence to the intervention (attendance to medical appointments with health care professionals and the control of cardiovascular risk factors). Kaplan Meier curves and propensity score-matched Cox regression models were used to evaluate the association between adherence and the incidence of CVOs.

**Results:**

A total of 52,507 patients were included. After propensity score matching, a sample of 35,574 patients was analyzed. Mean (SD) exposure time was 1.97 (0.92) years. Being adherent to the program was associated to a 85.4, 71.9, 32.4 and 78.9% risk reduction of in the low (HR 0.14; 95% CI 0.05–0.37; *p* < 0.001), medium (HR 0.28; 95% CI 0.21–0.36; *p* < 0.001), high-risk with DM (HR 0.67; 95% CI 0.43–1.04; *p* = 0.075) and hig-risk without DM (HR 0.21; 95% CI 0.09–0.48; p < 0.001) categories, respectively.

**Conclusions:**

The DTC program is effective in the reduction of the risk of CVOs. Population-based interventions may be an important strategy for the prevention of CVOs in underserved populations in the context of LMICs. A more exhaustive emphasis on the control of diabetes mellitus should be considered in these strategies.

## Introduction

Cardiovascular diseases (CVDs) are the leading cause of death globally [1]. It is estimated that nearly 75% of CVD-related deaths occur in low-and middle-income countries (LMICs) and an increase in their frequency is expected in the subsequent years. Type two diabetes mellitus (DM) is considered an independent risk factor for the occurrence of cardiovascular outcomes (CVO) such as stroke, myocardial infarction (MI), and congestive heart failure (CHF) in the context of the developing world (Hazard Ratio 1.69; 95% CI 1.43–2.00) [2]. Previous research suggests that although highly preventable, suboptimal blood pressure, blood glucose, total cholesterol, and Body Mass Index (BMI) contribute to the 63% of global mortality from CVDs, chronic kidney disease (CKD), and DM [3].

The control of cardiovascular risk factors is considered the cornerstone in the reduction of the incidence of CVO [4, 5]. However, the execution of population-based interventions in health care systems with budgetary constraints remains an important challenge [6]. The lack of real-world evidence regarding the effectiveness of interventions in these settings has resulted in the incorporation of practice guidelines developed in high-income countries (HIC) even though they may not be equally effective in LMICs due to vast differences in settings and patients [7]. It is recognized that although markers of poor socioeconomic status such as low-educational level are associated with greater exposure to the most common risk factors for CVDs across the different regions of the world, their effect is more pronounced in LMICs [8]. Adverse living conditions and unprepared health care systems also can influence the adherence of patients to interventions for CVD prevention and thus strongly predict the extent in which these interventions can achieve their intended goals [9, 10]. Therefore, the evaluation of the effect of interventions aimed at the prevention of CVOs in the context of LMICs using real-world data is required [7, 11].

Colombia is a predominantly urban country (76%) of over 50 million inhabitants where CVD accounts for 32,3% of total deaths per year [12, 13]. Nearly 50% of the population is affiliated to the health care system through governmental subsidies and a considerable proportion of this population resides in the Caribbean region of the country. The objective of this study was to evaluate the effectiveness of a cardiovascular risk management program (*De Todo Corazón - DTC* program) in the incidence of the first CVO in a low-income population from the Caribbean region of Colombia using adherence as the main variable of exposure.

## Methods

### Study design and setting

This was a propensity score-matched retrospective cohort study that used prospectively collected patient-level data of the population of patients enrolled in the DTC program of Mutual Ser. Mutual Ser is one of the largest health insurance companies in Colombia and has almost 1.4 million affiliates distributed in five departments of the Caribbean region of the country (Atlántico, Bolívar, Córdoba, Magdalena, and Sucre). The majority of affiliated patients have granted access to the benefits package of the Colombian Health Care System through governmental subsidies (i.e., subsidized regime). The effectiveness of the DTC program on the incidence of the first fatal or non-fatal CVO was evaluated by comparing incident cases between adherent and non-adherent patients to the program activities through a time-to-event analysis. As the DTC program is targeted at individuals based on their cardiovascular risk at enrollment, we stratified the study sample in four different groups according to their estimated baseline Framingham Risk Score (low risk, medium risk, high risk without DM, and high risk with DM). Time-to-event analyses were further conducted for adherent and non-adherent patients within each risk group over the time of exposure to the program interventions. Propensity-score matched samples from these groups were retrieved and analyzed to account for potential confounding variables that could produce biased effectiveness estimations. The STROBE guidelines for reporting cohort studies were followed [14].

### Participants

**Fig. 1:**
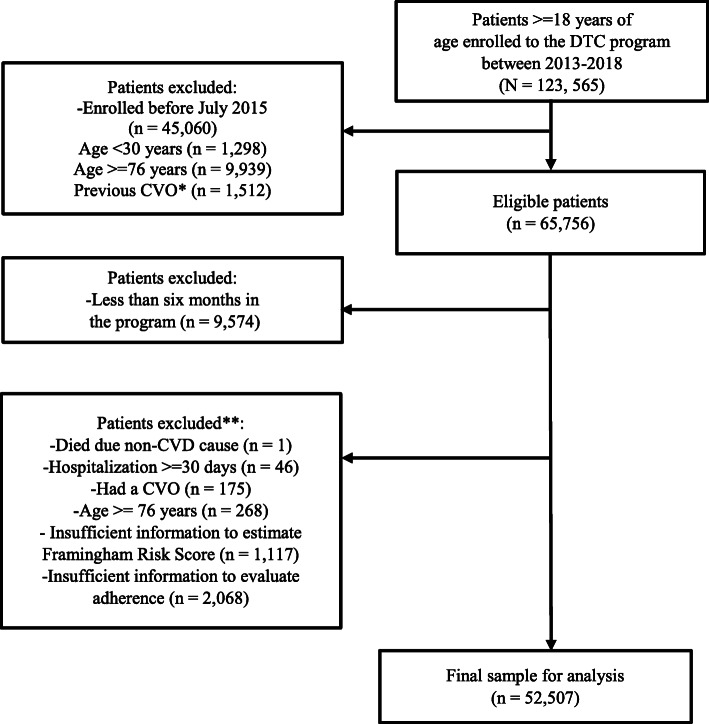
Patient identification and selection process. CVO = cardiovascular outcome, CVD = cardiovascular disease. *Patients with a previous CVO were excluded for the population of patients with an age between 30 to 75 years and enrolled in the program after July 01–2015. **The exclusion criteria were applied to the eligible patients with more than six months in the program

Adult patients (> = 18 years) with a diagnosis of hypertension (HTA), DM, CKD, or dyslipidemia affiliated to the DTC program between 2013 and 2018 were considered as the study population. Individuals with 30 to 75 years, enrolled between July 01–2015 and July 01–2018, and without a history of CVOs at enrollment were considered eligible. Among these, patients with less than 6 months of follow-up time in the program were excluded. Patients were also excluded if they experienced any of the following events in the first 3 months after the date of enrollment: a CVO, a prolonged hospitalization (≥ 30 days), died due to a non-CVD-related cause or reached an age of 76 years, whichever occurred first (Fig. 1).

### Data sources

The primary data source was an administrative database of the open cohort of patients enrolled in the DTC Program between January 2013 and December 2018. Patient-level data on demographic (i.e. age, sex), clinical and anthropometric (i.e., body weight, height, serum glucose, total cholesterol, high-density lipoprotein cholesterol [HDL-C], triglycerides, low-density lipoprotein cholesterol [LDL-C], blood pressure (BP), glycated hemoglobin (hba1c), glomerular filtration rate, ICD-10 diagnoses), and epidemiological (i.e., self-reported frequency of physical activity, smoking status, place of residence, and family history of HTA and DM) variables recorded in the outpatient setting by health care professionals at each medical visit was available for the study.

The frequency of CVOs between the time of exposure (July 01–2015 and December 31–2018) was retrieved from the all-cause consolidated hospitalization database of Mutual Ser. Incident cases of stroke, MI and CHF, were identified if an associated ICD-10 code was registered at discharge (stroke = I69, I67, I66, I65, I64, I63, G46, G45, I68; MI = I25, I24, I23, I22, I21, I20; CHF = I50, I13, I11). Information regarding the date and place of occurrence of the hospitalization event, length of stay, and vital status at discharge was also available for the study. Only records of deaths and CVOs in the inpatient setting were available for the study.

### Outcomes

The primary outcome of interest was the reduction in the risk of the first incident CVO between adherent and non-adherent patients to the program activities within each cardiovascular risk category over the time of exposure. Secondary outcomes were the crude incidence rates of CVOs for each risk group (using only the first observed event as the numerator and the total person-time years as the denominator) and the time from enrollment to CVO onset.

### Assessment of adherence

The adherence to the program was evaluated based on the observed frequency of medical appointments with health care professionals for clinical follow-up (general or specialized physicians and nurses, nutritionists, and psychologists), and the control of cardiovascular risk factors. The criteria defined for considering a patient as an adherent to the program activities were evaluated according to the cardiovascular risk stratification as follows: > = 2 visits to the general physician or nurses for the low-risk group, and > =4 and > =6 visits to general or specialized physicians and nurses for the medium and high-risk with and without DM groups, respectively. A general criterion of at least one visit to nutritionists and psychologists was defined.

Regarding the control of cardiovascular risk factors, the following criteria were evaluated equally for all patients in the study sample as follows: mean systolic BP/diastolic BP < 140/90 mmHg for patients with HTA, mean hba1c < 7% for patients with DM or HTA + DM, mean LDL-C < =100 mg/dL, a reported frequent or occasional history of physical activity or being non-smoker in all medical visits. A patient was considered adherent to the program if at least four of the main criteria were met (80%). Patients with insufficient information for the evaluation of adherence were excluded.

### The “*De todo corazón*” program

The DTC program is a structured evidence-based intervention focused on the primary prevention of CVOs. Patients are enrolled based on pre-defined screening criteria and are treated by different health care professionals according to the estimated cardiovascular risk at baseline (Framingham Risk Score). A minimum recommended frequency of health care services per year is offered through different private health care companies located in each department where the program is offered (Supplementary material 1). Interventions include pharmacological treatment, appointments with general physicians and nurses trained in cardiovascular health, specialized physicians, nutritionists, psychologists, and educational sessions focused on the adoption of healthy lifestyle habits. Mutual Ser uses a performance-based contract scheme in which health care companies receive financial incentives for achieving specific goals of cardiovascular risk factors control and frequency of CVOs in the enrolled population.

### Statistical analysis

Patient characteristics at baseline were summarized descriptively. Quantitative variables were summarized with means and standard deviations and categorical variables with absolute and relative frequencies. Baseline values were retrieved by selecting the first observation of these variables after the date of enrollment. Differences in patient characteristics between adherent and non-adherent patients before and after matching were assessed using absolute standardized mean differences (SMD).

We used the nearest neighbor propensity score matching method with a 3:1 optimal ratio to reduce selection bias and correct for possible imbalances between adherent and non-adherent patients within each risk group [15–17]. Multiple logistic regression models were used to obtain a set of propensity scores for each risk category using the adherence status variable as the outcome. The stepwise backward selection method was used to select relevant predictors of adherence. Sex, age, BMI, Systolic BP, Diastolic BP, LDL-C, HDL-C, total cholesterol, triglycerides, stage of renal function based on the glomerular filtration rate, and the department of residence were considered as potential predictors.

The non-parametric Kaplan-Meier survival method was used to evaluate differences in the probability of remaining CVO-free through the exposure time between the matched samples of adherent and non-adherent patients within each risk group. Patients were censored if after the first 3 months of exposure to the program they experienced a prolonged hospitalization (≥ 30 days), died due to a non-CVD-related cause, reached the age of 76 years, or the study period ended, whichever occurred first.

Cox Proportional Hazard models were used to evaluate the association between the adherence and incidence of CVOs by the estimation hazard ratios (HRs) of the first CVO between adherent and non-adherent patients through the study period. A two-sided *p*-value < 0.05 was considered statistically significant. The R statistical package version 3.4.3 was used for data management and analysis (R Core Team, R Foundation for Statistical Computing).

## Results

### Sample characteristics

**Fig. 2:**
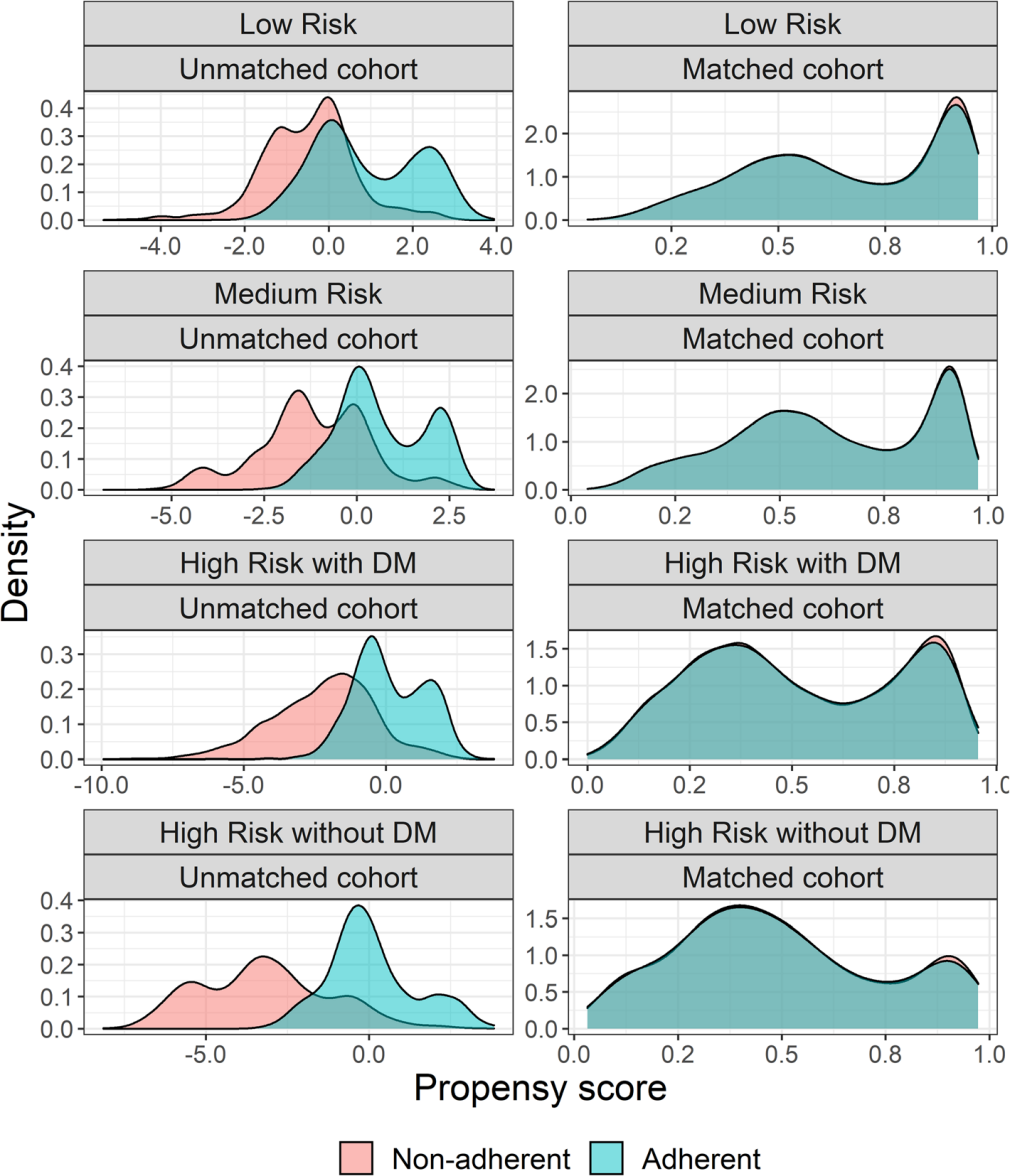
Propensity score distribution before and after matching for each cardiovascular risk group

The identification and selection process of the study sample is shown in Fig. 1. A final sample of 52,507 patients was obtained. Baseline characteristics of the matched and unmatched samples by adherence status are shown in Table 1. After propensity score matching, a sample of 35,574 patients was analyzed and the baseline characteristics of the adherent and non-adherent patients within each cardiovascular risk category were balanced (Fig. 2) (See Supplementary material 2 for further details). Mean (SD) exposure time of the analyzed sample was 1.97 (0.92) years, with a minimum and maximum time of 6.24 and 3.49 years, respectively.

**Table 1:** Sample characteristics at baseline by adherence status

Characteristic	Unmatched^a^			Matched^a^		
	Adherent	Non-adherent	SMD	Adherent	Non-adherent	SMD
**Frequency of patients, n (%)**	20,613	31,894		19,943	15,631	
**Sex**						
Female	13,914 (67.5)	19,983 (62.7)	0.103	13,464 (67.5)	10,351 (66.2)	0.032
Male	6699 (32.5)	11,911 (37.3)	0.103	6479 (32.5)	5280 (33.8)	0.032
**Age in years, mean (SD)**	56.1 (10.2)	56.4 (10.4)	0.034	56.1 (10.2)	56.2 (10.4)	0.011
**Overweight or obesity (BMI > 25)**	13,356 (64.9)	21,023 (66)	0.024	13,001 (65.2)	10,337 (66.1)	0.004
**Diagnosis at baseline**						
Hypertension	19,796 (96)	30,010 (94.1)	0.1	19,179 (96.2)	14,904 (95.3)	0.074
Diabetes millitus type 2	4224 (20.5)	10,983 (34.4)	0.345	4061 (20.4)	4033 (25.8)	0.135
Dyslipidemia	4759 (23.1)	13,962 (43.8)	0.491	4681 (23.5)	5084 (32.5)	0.075
Chronic kidney disease	2326 (11.3)	3226 (10.1)	0.037	2252 (11.3)	1560 (10)	0.003
**Prevalence groups**						
HTA	10,733 (52.1)	9074 (28.5)	0.473	10,353 (51.9)	6542 (41.9)	0.038
HTA + Dyslipidemia	3665 (17.8)	9329 (29.3)	0.3	3603 (18.1)	3782 (24.2)	0.086
HTA + DM	2432 (11.8)	5037 (15.8)	0.124	2340 (11.7)	2202 (14.1)	0.102
HTA + DM + Dyslipidemia	1582 (7.7)	1432 (4.5)	0.12	1523 (7.6)	864 (5.5)	0.015
HTA + CKD	817 (4.0)	1884 (5.9)	0.1	764 (3.8)	727 (4.7)	0.074
DM	640 (3.1)	3344 (10.5)	0.425	631 (3.2)	818 (5.2)	0.034
HTA + CKD + Dyslipidemia	409 (2.0)	1076 (3.4)	0.1	403 (2.0)	410 (2.6)	0.044
HTA + DM + CKD	290 (1.4)	505 (1.6)	0.015	282 (1.4)	212 (1.4)	0.002
HTA + DM + Dyslipidemia + CKD	45 (0.2)	213 (0.7)	0.096	44 (0.2)	74 (0.5)	0.036
**Cardiovascular risk factors, mean (SD)**						
Systolic blood pressure (mmHg)	127.1 (14.9)	129.1 (17.2)	0.134	127.1 (14.9)	128.2 (16.3)	0.07
Diastolic blood pressure (mmHg)	80.2 (11)	81.5 (12.1)	0.12	80.3 (11)	81 (12.2)	0.054
Glycated hemoglobin (HbA1c)	6.9 (1.8)	8.4 (2.4)	0.801	6.9 (1.7)	7.2 (1.7)	0.036
Total cholesterol (mg/dl)	190.5 (40.9)	201.2 (44.1)	0.263	190.2 (40.4)	194.1 (41.4)	0.019
LDL cholesterol (mg/dl)	109.7 (32.8)	117.8 (33.3)	0.247	109.8 (32.8)	113.1 (31.7)	0.016
HDL cholesterol (mg/dl)	47.7 (11.1)	47.6 (10.9)	0.011	47.7 (11.1)	47.6 (11)	0.018
Triglycerides (mg/dl)	153.1 (72.4)	169.6 (79.4)	0.229	152.3 (71.3)	159.6 (72.2)	0.045
Current smoking^b^	1075 (5.2)	8525 (26.7)	0.968	1050 (5.3)	1384 (8.9)	0.006
**Frequency of physical activity**						
Never	9172 (44.5)	25,381 (79.6)	0.706	9050 (45.4)	11,196 (71.6)	0.024
Occasional	7396 (35.9)	4350 (13.6)	0.464	7098 (35.6)	2868 (18.3)	0.049
Frequent	4045 (19.6)	2163 (6.8)	0.323	3795 (19.0)	1567 (10.0)	0.029

^a^The overall matched and unmatched samples equal to the sum of the marched and unmatched patients within each risk group. ^b^A patient was considered a current smoker if reported to be a smoker or ex-smoker during the last year in the program. *BMI* Body Mass Index, *LDL* Low-density lipoprotein cholesterol, *HDL* High density lipoprotein cholesterol, *HTA* Hypertension, *DM* Diabetes Mellitus, *SMD* Absolute Standardized Mean Difference

### Adherence to the program

A total of 20,613 patients (39.2%) were considered adherents to the program. The highest proportion of adherent patients was found in the low-risk group (53.9%) followed by the medium (43.8%) and high-risk groups (diabetic = 27.7% and non-diabetic = 18.9%, respectively). Non-smoking status, controlled blood pressure, and visits to nurses and general or specialized physicians were the most frequently meet adherence criteria. Only 41.2% of patients in the unmatched sample had a mean hba1c < 7% in the study period. The relative frequency of patients in the matched and unmatched samples meeting each adherence criterion according to clinical diagnosis and cardiovascular risk category is shown in the Supplementary material 3.

### Frequency of cardiovascular outcomes

A total of 879 (1.6%) patients in the unmatched sample experienced any CVO during the time of exposure (July 01–2015 and Dec 31–2018). Of these, 44.1% (374), 33.6% (285), and 22.2% (188) had a MI, stroke, and CHF diagnosis at discharge, respectively. A total of 32 patients had more than one CVO diagnosis (0.1%). After propensity-score matching, 560 CVOs were observed in the sample obtained. A similar distribution of events between the unmatched and matched samples was found with AMI (47.8%) as the most frequent CVO, followed by stroke (33.2%) and CHF (22.6%). The overall rates of any CVO per 1000 person-years were 8.45 (95% CI 7.90–9.03) and 8.11 (95% CI 7.45–8.81) in the unmatched and matched samples.

According to cardiovascular risk, the rates of CVOs per 1000 person-years were 4.08 (95% CI 3.13–5.22), 8.59 (95% CI 7.82–9.41), 8.66 (95% CI 7.64–9.78), and 18.93 (95% CI 15.37–23.07) in the low-risk, medium-risk, high-risk with DM and high-risk without DM groups, respectively. A similar trend in rates were observed in the matched sample with an overall rate of 8.11 (7.45–8.81) CVOs per 1000 person year and 3.99 (95% CI 2.97–5.25), 9.07 (95% CI 8.15–10.0), 7.58 (95% CI 6.28–9.07) and 21.86 (95% CI 15.39–30.13) in the low-risk, medium-risk, high-risk with DM and high-risk without DM groups, respectively.

### Frequency of cardiovascular outcomes according to adherence status

The frequency of CVOs according to risk group and adherence status for the matched and unmatched samples are shown in Table 2. The overall rates of the first CVO per 1000 person-years were 4.70 (95% CI 4.04–5.42) and 10.74 (95% CI 9.96–11.7) for adherent and non-adherent patients in the unmatched sample. After propensity score matching, the overall rates were 4.71 (95% CI 4.04–5.45) and 15.88 (95% CI 14.46–17.40) for adherent and non-adherent patients.

**Table 2:** Frequency of cardiovascular outcomes according to the cardiovascular risk group and adherence status for the matched and unmatched samples

Risk group	Adherence status	Person-years	Frequency of patients	Frequency of events	Rate	95% CI	
						Lower bound	Upper bound
**Matched sample**							
High Risk with DM	Adherent	7855	4061	47	6,0	4,4	8,0
	Non-adherent	7450	4050	70	9,4	7,3	11,8
High Risk without DM	Adherent	876	508	7	8,0	3,2	16,5
	Non-adherent	755	537	32	42,9	29,4	60,5
Medium Risk	Adherent	22,120	11,509	114	5,2	4,3	6,2
	Non-adherent	15,709	8650	294	18,7	16,7	21,0
Low Risk	Adherent	7396	3865	12	1,6	0,8	2,8
	Non-adherent	4962	2471	62	12,5	9,6	16,0
**Unmatched sample**							
High Risk with DM	Adherent	8092	4224	48	5,9	4,4	7,9
	Non-adherent	21,925	10,983	212	9,7	8,4	11,1
High Risk without DM	Adherent	934	541	7	7,5	3,0	15,4
	Non-adherent	4241	2323	91	21,5	17,3	26,3
Medium Risk	Adherent	22,591	11,797	114	5,0	4,2	6,1
	Non-adherent	30,711	15,123	344	11,2	10,0	12,4
Low Risk	Adherent	7743	4051	16	2,1	1,2	3,4
	Non-adherent	7694	3465	47	6,1	4,5	8,1

*CI* Confidence Interval, *DM* Diabetes Mellitus

### Effect of the DTC program in the risk of cardiovascular outcomes

**Fig. 3:**
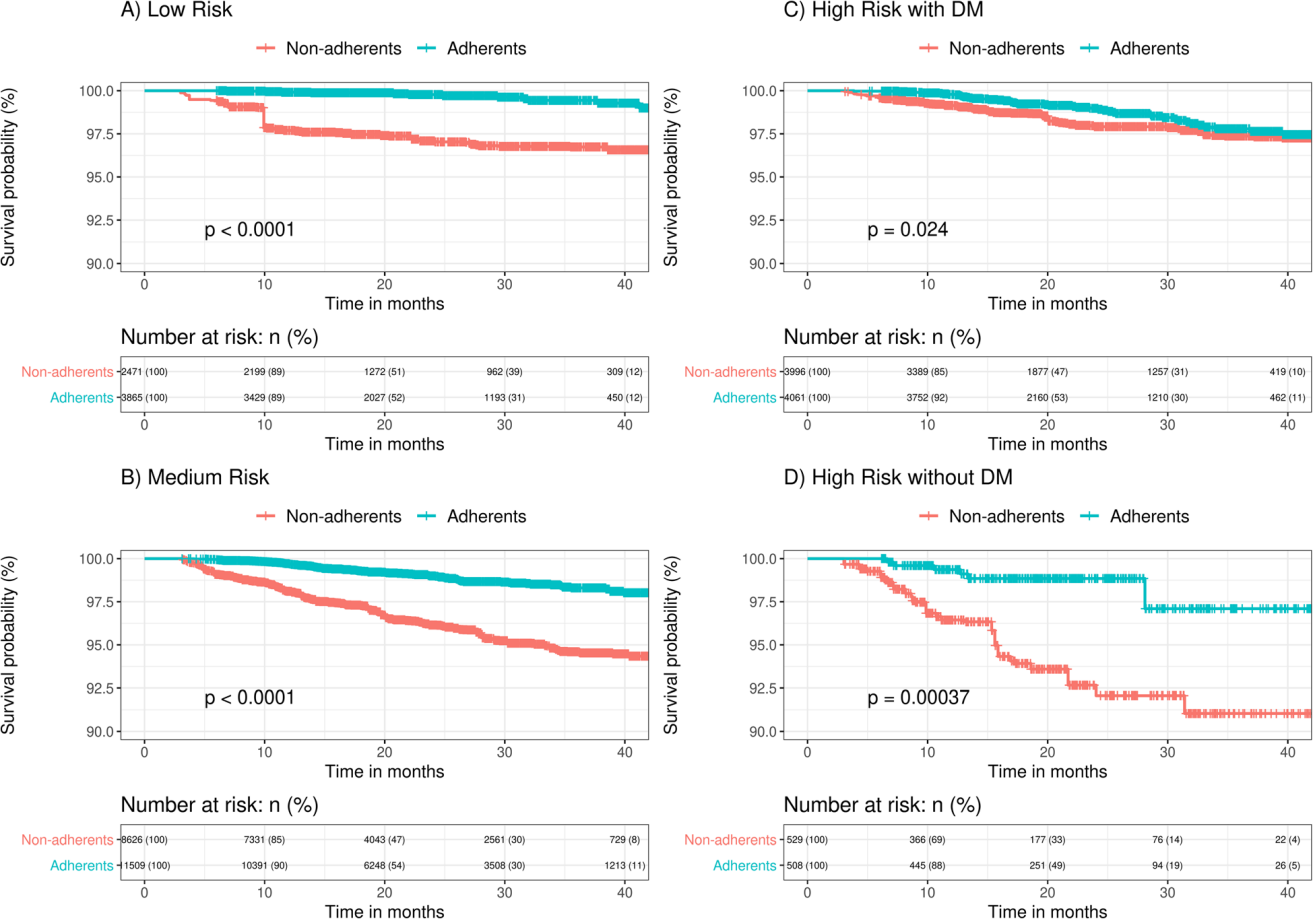
Survival curves of any CVO according to adherence status for each cardiovascular risk group

Weighted Kaplan-Meier survival curves describing the proportion of patients without CVOs over the study period for adherent and non-adherent patients in each cardiovascular risk group are shown in Fig. 3. The Cox Proportional Hazard model showed a 68.9% reduction in the risk of the first CVO in adherent patients compared to non-adherent patients in the overall sample (HR 0.31; 95% CI 0.24–0.38; *p* < 0.001). According to risk group, being adherent to the program was associated to a 85.4, 71.9, 32.4 and 78.9% risk reduction of in the low (HR 0.14; 95% CI 0.05–0.37; p < 0.001), medium (HR 0.28; 95% CI 0.21–0.36; p < 0.001), high-risk with DM (HR 0.67; 95% CI 0.43–1.04; *p* = 0.075) and hig-risk without DM (HR 0.21; 95% CI 0.09–0.48; p < 0.001) categories, respectively.

## Discussion

To our knowledge, this is the first study that evaluated the effectiveness of a CVD prevention program in the incidence of CVOs using real-world data from a low-income population in Latin America. This analysis suggests that a comprehensive program aimed at the primary prevention of CVOs using multiple risk factor interventions can achieve an overall risk reduction of 68.9% in the risk of the first CVO. We also found a 85.4, 71.9, 32.4, and 78.9% risk reduction for patients with low, medium, and the high cardiovascular risk with and without DM, respectively. We consider that the non-significant effect of the program in the high-risk group with DM was associated with greater difficulty of the program in achieving metabolic control (~ 41.2%).

The effectiveness of multiple cardiovascular risk factors interventions has been previously evaluated through randomized trials. A previous systematic review by Ebrahim et al. that included 55 trials (163,471 participants) with a median follow-up time of 1 year (with a range of 6 months to 12 years) suggests that multiple risk factor interventions have no effect in CVD mortality or incidence in general populations but may be effective in high-risk hypertensive (OR 0.78 [95% IC 0.68–0.89]) and diabetic populations (OR 0.71 [95% CI 0.61 to 0.83]). However, these results may be representative of the effectiveness of these interventions in high-income countries, where the majority of studies were conducted [5].

A previous systematic review of RCTs by Uthman et al. in 2015 evaluated the effectiveness of multiple risk factor interventions aimed at the modification of risk factors for the prevention of CVD in LMICs. The authors highlight the lack of evidence of the effectiveness of interventions in these settings and suggest no effect in CVOs based on an RCT of 232 participants that reported a RR of 0.57 (95% CI 0.11 to 3.07) [18].

We consider that our study fills important gaps in the previous literature regarding the effectiveness of population-based multiple risk factor interventions in real-world settings in the context of LMICs. We performed a propensity score-matched study using a large cohort of adult patients with a previous diagnosis of HTA, DM, CKD, or dyslipidemia and adherence as the main variable of exposure. We consider that these are the major strengths of our study. Propensity score methods are considered robust quasi-experimental methodologies for the evaluation of treatment effects in observational studies and their use in cardiovascular research has increased recently [19].

Our study has limitations and our results should be viewed with caution. We were unable to include the prescription of medications in the assessment of adherence and this would be a source of uncertainty in our evaluation. However, we consider that our definition of adherence can be considered robust as we were able to account for multiple relevant criteria, such as attending to the program activities and control of cardiovascular risk factors. Another relevant limitation in our study was the restricted access to death records in other scenarios different from the hospitalization setting. Therefore, CVD and non-CVD related deaths that occurred at patient homes, for example, were not included in the time-to-event analysis, and this would potentially influence our estimations of the effectiveness of the DTC program. However, due to the strict follow-up strategy implemented in the program, we consider that the majority of expected deaths were observed through the hospitalizations database used in this study. We consider that these limitations would be overcome with more exhaustive data sources and a longer follow-up period.

## Conclusions

The DTC program is effective in the reduction of the risk of CVOs. Population-based interventions can be an important strategy for the prevention of CVO in low-income individuals in the context of the developing world. A more exhaustive emphasis on the control of diabetes mellitus should be considered in these strategies.

## Supplementary information

**Additional file 1 Supplementary material 1**. Frequency of health care services offered through the DTC program according to the cardiovascular risk group

**Additional file 2 Supplementary material 2**: Patient characteristics at baseline for matched and unmatched samples according to cardiovascular risk categories

**Additional file 3 Supplementary material 3**. Frequency of patients in the matched and unmatched samples meeting adherence criteria according to clinical diagnosis and cardiovascular risk category

## Data Availability

The analysis reported in this study uses patient-level data from the *De Todo Corazón* program administered by Mutual Ser. The patient-level data is not publicly available. The R code used for the construction of the analyzed sample and all statistical analyses can be available from the corresponding author on reasonable request.

## Abbreviations

CVDs: Cardiovascular diseases
LMICs: Low-and middle-income countries
CVO: Cardiovascular outcome
DM: Diabetes Mellitus
MI: Acute Myocardial Infarction
HTA: Hypertension
CHF: Congestive Heart Failure
BMI: Body Mass Index
HIC: High-income countries
DTC: “*De Todo Corazon*” Risk Management Program
IRR: Incidence rate ratio
