# Supplementary material for: Effectiveness of a prevention program in the incidence of cardiovascular outcomes in a low-income population from Colombia: a real-world propensity score-matched cohort study
Source: BMC Public Health. 2020 Sep 17;20:1416. doi: 10.1186/s12889-020-09516-5 (PMC7500541; doi:10.1186/s12889-020-09516-5)
Supplement: Supplementary file 1 — Additional file 1 Supplementary material 1. Frequency of health care services offered through the DTC program according to the cardiovascular risk group [file 12889_2020_9516_MOESM1_ESM.docx]

Supplementary material 1. Minimum recommended frequency of health care services offered through the DTC program according to cardiovascular risk group

| Health care service | Risk group* | | | |
| --- | --- | --- | --- | --- |
|  | Low risk hypertensive patients | Medium risk hypertensive patients | High risk hypertensive patients | High risk hypertensive and diabetic patients |
| Blood test | 1 | 1 | 1 | 1 |
| Glycemic test | 1 | 1 | 1 | 4 |
| Lipid profile test | 1 | 1 | 1 | 1 |
| Urinalysis | 1 | 1 | 1 | 1 |
| Creatinine test | 1 | 3 | 3 | 3 |
| Electrocardiogram test | 1 | 1 | 1 | 1 |
| Microalbuminuria test |  |  | 1 | 1 |
| Creatinine blood test |  |  | 1 | 1 |
| Glycated hemoglobin test |  |  |  | 4 |
| General physician visit | 2 | 4 | 6 | 6 |
| Internal medicine visit |  | 1 | 4 | 4 |
| Nutritionist visit | 1 | 1 | 4 | 4 |
| Nursery visit | 4 | 4 | 6 | 6 |
| Educational talks | 6 | 6 | 6 | 6 |

*Estimated risk is routinely assessed with the Framingham Risk Score
