# Supplementary material for: Effectiveness of a prevention program in the incidence of cardiovascular outcomes in a low-income population from Colombia: a real-world propensity score-matched cohort study
Source: BMC Public Health. 2020 Sep 17;20:1416. doi: 10.1186/s12889-020-09516-5 (PMC7500541; doi:10.1186/s12889-020-09516-5)
Supplement: Supplementary file 3 — Additional file 3 Supplementary material 3. Frequency of patients in the matched and unmatched samples meeting adherence criteria according to clinical diagnosis and cardiovascular risk category [file 12889_2020_9516_MOESM3_ESM.docx]

Supplementary material 3. Frequency of patients in the matched and unmatched samples meeting adherence criteria according to clinical diagnosis and cardiovascular risk category

| **Cardiovascular risk group** | **Adherence criteria** | **Proportion of patients meeting each adherence criterion** | |
| --- | --- | --- | --- |
|  |  | **Unmatched** | **Matched** |
| All | Visits to general or specialized physicians and nurses | 46,815(89.2%) | 32,325(90.8%) |
|  | Visits to nutritionists and psychologists | 32,885(62.6%) | 22,760(64%) |
|  | Mean systolic BP/diastolic BP<140/90 mmHg | 46,931(89.4%) | 32,054(90.1%) |
|  | Mean LDL-C<=100 mg/dL | 17,439(33.6%) | 13,328(37.5%) |
|  | Mean hba1c <7% | 6,266(41.2%) | 4,573(56.5%) |
|  | Being non-smoker a in all medical visits | 42,907(81.7%) | 33,151(93.1%) |
|  | Self-reported frequent or occasional physical activity | 17,954(34.2%) | 15,324(43.1%) |
| High Risk with DM | Visits to general or specialized physicians and nurses | 13,012(85.6%) | 7,127(88.1%) |
|  | Visits to nutritionists and psychologists | 10,472(68.9%) | 5,786(71.5%) |
|  | Mean systolic BP/diastolic BP<140/90 mmHg | 14,038(92.3%) | 7,495(92.7%) |
|  | Mean LDL-C<=100 mg/dL | 5,452(36.7%) | 3,347(41.4%) |
|  | Mean hba1c <7% | 6,266(41.2%) | 4,573(56.5%) |
|  | Being non-smoker a in all medical visits | 12,249(80.5%) | 7,534(93.1%) |
|  | Self-reported frequent or occasional physical activity | 5,085(33.4%) | 3,909(48.3%) |
| High Risk without DM | Visits to general or specialized physicians and nurses | 2,367(82.6%) | 867(83.9%) |
|  | Visits to nutritionists and psychologists | 1,793(62.6%) | 671(65%) |
|  | Mean systolic BP/diastolic BP<140/90 mmHg | 2,170(75.8%) | 795(77%) |
|  | Mean LDL-C<=100 mg/dL | 636(22.4%) | 263(25.7%) |
|  | Being non-smoker a in all medical visits | 857(29.9%) | 533(51.6%) |
|  | Self-reported frequent or occasional physical activity | 1,018(35.5%) | 715(69.2%) |
| Low Risk | Visits to general or specialized physicians and nurses | 7,225(96.1%) | 6,127(96.7%) |
|  | Visits to nutritionists and psychologists | 4,246(56.5%) | 3,757(59.3%) |
|  | Mean systolic BP/diastolic BP<140/90 mmHg | 6,989(93%) | 5,917(93.4%) |
|  | Mean LDL-C<=100 mg/dL | 3,182(42.7%) | 2,868(45.3%) |
|  | Being non-smoker a in all medical visits | 7,317(97.4%) | 6,261(98.8%) |
|  | Self-reported frequent or occasional physical activity | 2,528(33.6%) | 2,321(36.6%) |
| Medium Risk | Visits to general or specialized physicians and nurses | 24,211(89.9%) | 18,204(90.4%) |
|  | Visits to nutritionists and psychologists | 16,374(60.8%) | 12,546(62.3%) |
|  | Mean systolic BP/diastolic BP<140/90 mmHg | 23,734(88.2%) | 17,847(88.7%) |
|  | Mean LDL-C<=100 mg/dL | 8,169(30.5%) | 6,850(34.1%) |
|  | Being non-smoker a in all medical visits | 22,484(83.5%) | 18,823(93.5%) |
|  | Self-reported frequent or occasional physical activity | 9,323(34.6%) | 8,379(41.6%) |

*Relative frequencies are reported using the total frequency of patients within each risk group as denominator. DM = Type 2 Diabetes mellitus, BP = Blood pressure, LDL-C = Low-density cholesterol
